# Determination of Technological Parameters and Characterization of Microbiota of the Spontaneous Sourdough Fermentation of Hull-Less Barley

**DOI:** 10.3390/foods10102253

**Published:** 2021-09-23

**Authors:** Sanita Reidzane, Zanda Kruma, Jekaterina Kazantseva, Anna Traksmaa, Dace Klava

**Affiliations:** 1 Faculty of Food Technology, Latvia University of Life Sciences and Technologies, Riga Street 22, LV-3004 Jelgava, Latvia; zanda.kruma@llu.lv (Z.K.); dace.klava@llu.lv (D.K.); 2 Center of Food and Fermentation Technologies, Akadeemia tee 15a, 12618 Tallinn, Estonia; jekaterina@tftak.eu (J.K.); anna@tftak.eu (A.T.); 3 Department of Chemistry and Biotechnology, Tallinn University of Technology, Akadeemia tee 15, 12618 Tallinn, Estonia

**Keywords:** sourdough, fermentation, hull-les barley, microbial community dynamics

## Abstract

The development of microorganisms of sourdough and biodiversity of microbiota can be influenced by changing the parameters of the technological process such as the ratio of flour and added water, the fermentation temperature and time. The Box–Behnken design methodology was used to determine the optimal parameters for the three-phase spontaneous backslopping fermentation process of hull-less barley sourdough, as well as to characterize the microbiological diversity. The optimized parameters of backslopping fermentation are flour and water ratio 1:1.13, temperature 30 °C, time 24 h in the 1st backslopping; the inoculate, flour and water ratio 1:1:1.3, temperature 31 °C, time 14 h in the 2nd backslopping, and the inoculate, flour and water ratio 1:1:1.5, and temperature 28.5 °C, time 12 h in the 3rd step of backslopping. In the controlled spontaneous fermentation environment in three backslopping steps, the microbiological research of hull-less barley sourdough has confirmed the dominance of species *Pediococcus pentosaceus* in the 3rd backslopping step of spontaneous fermentation. The developed spontaneous hull-less barley sourdough is consistent with the number of lactic bacteria and yeasts in line with that seen by the active sourdough.

## 1. Introduction

Barley (*Hordeum vulgare* L.) as cereal is more used for production of beer, but in the last few years, there is a growing interest to use it as food—like breakfast cereal or groats. The interest in using hull-less barley is growing because it is easy to use (there is no hull) and has high nutrition value and high content of fibers, β-glucans and arabinoxylans [1,2]. Barley is not used in bread baking especially because of the properties of β-glucans, since barley has bad baking performance and sensory quality as bread. However, the research shows that the use of a sourdough system in making bread, especially wholemeal bread, creates a positive effect [3,4]. Also, during the fermentation, the fibers become more soluble, and this can have a positive effect on the health of people [5]. By using wholegrain flour, a more diverse environment is created for the growth of microorganisms, their development and metabolic activity. The available amount of vitamins, minerals, fibers and biologically active components influences the microbiota of the sourdough [6].

There are descriptions of different types of sourdough fermentation: spontaneous fermentation, fermentation with a starter culture and dried/stabilized after preparation sourdough [7], together with mixed-type fermentation that has been started with pure culture and then succeeding continuing with backslopping procedure (also called “refreshment”) [8]. Each of these types is characterized by a different combination of microorganisms.

Sourdough fermentation can start spontaneously not only with starter culture or mother dough. In spontaneous fermentation, the natural microbiota of the flour is used, and it is influenced by the microbiota of the environment and raw materials. The sourdough prepared with the spontaneous method is based on backslopping processes—repeated cyclic backslopping with a new amount of water and flour [9]. Through the cycles of backslopping, the microorganisms from different genera have adapted depending on available nutrition elements and influencing parameters of the technological process: temperature, time and amount of added water [10]. The traditional spontaneous sourdough or the type 1 sourdough is used in the artisan bakeries: the time of fermentation is from short to average (6 to 24 h), the temperature is moderate, 20–30 °C, with low DY < 200 (DY = (flour + water) × 100/flour weight) [8,11]. The type 1 sourdough is suitable for leavening the dough without additional leavening agents [7].

There are several protocols to start spontaneous sourdough preparation, the better-known ones are the French system for wheat sourdough, the U.S sourdough from wheat and/or rye flour and the spontaneous rye sourdough preparation, which is traditional for the Northern and Eastern part of Europe [7]. In Latvia, the traditional rye bread is made from mother’s dough, which is adapted in bakeries [12]. Spicher and Stephan [13] describe different 3–4 step (Stufe) systems to start fermentation with different parameters from 68 to 96 h, in all steps together, at 23–35 °C, and 160–400 DY. Kozlinskis [12] describes the three-phase system of preparing the rye sourdough. A fermentation system with several backslopping steps allows the option of slowly reducing the acidity as the result of activity of the homofermentative lactic acid bacteria in the sourdough and providing a favorable environment for the growth of yeasts and heterofermentative lactic acid bacteria. In this way, the function of type 1 sourdough leavening would be secured. At the end of the last step of fermentation, such sourdough can be seen as a starter culture, which contains diverse microbiota [7]. But there is still little research about the use of other cereals, especially barley, for preparation of spontaneous sourdough.

To obtain an active and complete type 1 sourdough from wheat or rye, at least three backslopping steps are necessary [8,14]. Stable ripe active sourdoughs are characterized by high carbohydrate concentrations, low pH, and higher lactic acid bacteria cell counts >10^8^ CFU g^−1^ (colony forming units per gram) compared to the yeast cell counts <10^7^ CFU g^−1^ [8]. The pH of the well-developed sourdough is in the range of 3.5–4.3 [7]. Several influencing parameters in this process can be changed in order to achieve the desired result, such as the ratio of flour and water (DY), temperature and time [8,15].

The traditional cereals used for spontaneous fermentation such as rye and wheat are mainly described and researched, but barley can be also used as an alternative. The fermentation capacity of sourdough is determined not only by the microbiota of the flour but also by the type of flour that is used (cereal culture, level of milling, activity of enzymes). The β-glucans in the hull-less barley are characterized by the high capacity to absorb water, and this aspect might be considered as an influencing factor in the making of spontaneous sourdough, which helps for successful development of needed microbiota.

In the active sourdoughs of barley, typical diverse cereal microbiota are observed. The barley sourdough with the fermentation parameter characteristics for type 1 sourdough contains *Pediococcus* (dominant species), *Lactobacillus curvatus* (new name *Latilactobacillus curvatus* [16]), *Leuconostoc mesenteroides* [17], *Lactobacillus plantarum* (new name *Lactiplantibacillus plantarum* [16,18])*, Weisella confusa* [10] bacteria, and *Saccharomyces cerevisiae, Candida humilis*, and *Pichia anomala* yeast species [9]. The main role of lactic acid bacteria is acidifying, but yeasts secure leavening by relieving CO_2_ [19]. From this aspect, it is important to develop symbiosis between lactic acid bacteria and yeasts. By changing the influencing parameters, it is possible to determine which microbiota will develop. It is possible to achieve the dominance of heterofermentative or homofermentative microorganisms as well as the development or complete disappearance of yeasts.

There is just a little research about the development of spontaneous microbiota of hull-less barley [20]. However, it would be useful to research specifically the starting steps of the sourdough of spontaneous fermentation, as well as the optimization of the influencing parameters for the development of the desired ecosystem. A multistep production system of spontaneous sourdough with three backsloppings is used to apply the different optimal conditions for every step, which allows it to be used for specific applications, mainly as a leavening agent for sourdough bread production or as a mother sponge stored till being used for the next bread making stage.

Taking into account that the nature of the fermentation sourdoughs is very diverse and is characterized by simultaneous mutual interference of different factors, the response surface methodology would be the most appropriate method for optimizing the influencing factors [20,21].

The goal of the research is to determine the optimal fermentation parameters of the spontaneously fermented active sourdough in a controlled environment in the three phases of fermentation, and to characterize the diversity of microbiota depending on the conditions of fermentation in three stages of the development.

## 2. Materials and Methods

### 2.1. Raw Material

Grains of hull-less barley (Hordeum vulgare) cultivar ‘Kornelija’ from the harvest of 2020 grown in Stende (lat. 57.1412° N, long. 22.5367° E) were used in the study. Harvested grains were cleaned in a cleaner (PETKUS Technologie GmbH, Hamburg, Germany) with the rectangular holes sieve (2.2 × 20 mm). The whole grain flour of hull-less barley cultivar ‘Kornelija’ (moisture 12.05 ± 0.42%, protein 17.76 ± 0.26%, starch 57.84 ± 0.46%, soluble dietary fiber 23.3 ± 4.7%, insoluble dietary fiber 3.2 ± 0.8%) were obtained by milling the hull-less barley grains in a Hawos laboratory mill (Hawos Kornmühlen GmbH, Hamburg, Germany). Flour was sieved through AS 200 mesh (Retsch GmbH, Haan, Germany), particle size 160–710 µm, among which 50% were about 450 µm.

### 2.2. Experimental Design for Sourdough Preparation and Statistical Analysis

Wholemeal hull-less barley flour cultivar “Kornelija” was used. Sourdough was prepared under laboratory conditions (controlled conditions) in the Bread technology laboratory of the Faculty of Food Technology of the Latvia University of Life Sciences and Technologies.

The Box–Behnken design method was used in order to evaluate the simultaneous impact of independent factors (amount of added water, X_1_; temperature, X_2_; time of fermentation, X_3_) to the parameters (responses) (Table 1), like the pH, the number of lactic acid bacteria and yeasts.

Sourdough was prepared by mixing wholemeal barley flour and tap water. As the base for preparation of spontaneous sourdough, the three-stage strategy with one backslopping procedure for each fermentation step was used. To start the fermentation, 100 g hull-les barley flour and the amount of added water, fermentation time and temperature were taken as indicated in the experimental design (Table 1). In the 2nd and 3rd backslopping of sourdough preparation, 100 g of previously fermented sourdough was added, mixed with the amount of flour and water as specified in the design of the experiment (Table 1).

Next, the optimized parameters of the factors of fermentation steps were established in accordance with the defined criteria of each fermentation step.

Equation (1) is a second order polynomial equation:

(1) $$Y=\beta_{0}+\beta_{1}X_{1}+\beta_{2}X_{2}+\beta_{3}X_{3}+\beta_{4}X_{1}X_{2}+\beta_{5}X_{2}X_{3}+\beta_{6}X_{1}X_{3}+\beta_{7}X_{1}^{2}+\beta_{8}X_{2}^{2}+\beta_{9}X_{3}^{2}.$$

The minimum and maximum values of the factors influencing the spontaneous fermentation were established beforehand. Three Box–Behnken designs were created—one composite design with 15 runs for each step.

The values of the influencing factors were entered—chosen by Design-Expert version 12 (Statease Inc., Minneapolis, MN, USA). Statistical significance was measured by analysis of variance (ANOVA). To evaluate the adjustment of the models, regression coefficients, the *p*-values and determination coefficient *R*^2^ were considered.

### 2.3. Determination of Number of Lactic Acid Bacteria (LAB) and Yeasts and Statistical Analysis

Preparation of test samples, an initial suspension and decimal dilutions for microbiological examination were performed according to the ISO 6887-1:1999 [22].

The enumeration of lactic acid bacteria has been established according to the ISO 15214Standard [23], using the MRS agar (Biolife) selective medium. The Petri plates were incubated for 72 h at 30 °C in an incubator cabinet.

The yeast numbers have been established according to the ISO 21527-1 Standard [24], using the malt extract agar (Biolife). The Petri plates were incubated for 72 h at 25 °C.

The horizontal method for the enumeration of microorganisms, Colony-count technique according to EN ISO 4833:2003, was applied. In order to establish the number of the colonies of microorganisms, the automatic counter of colonies aColyte (Topac Inc., Cohasset, MA, USA) with error limit <5% has been used. The total number of microorganisms was showed as colony forming units log CFU g^−1^.

The data were subjected to one-way analysis of variance (ANOVA). Significant differences (*p* < 0.05) were determined using Tukey’s Post Hoc test.

### 2.4. Determination of pH

The pH of sourdough was determined according to the AACC 02-52:1999 method [25] using a pH meter (Mettler—Toledo GmbH, Giessen, Germany) at 20 ± 1 °C.

### 2.5. Statistical Analysis

Optimized factors in the three backslopping steps of fermentation and experimentally determined data of pH, LAB and yeasts enumeration were subjected to one-way analysis of variance (ANOVA), and significant differences (*p* < 0.05) were determined using Tukey’s Post Hoc test.

### 2.6. Microorganisms Identification

After each step of sourdough fermentation in each step, the samples were lyophilized in freeze-dryer FT33 (Armfield Ltd., Hampshire, UK) in a condenser chamber at 40 °C and 6.4 Pa for 72 h.

For microbial DNA extraction, 5 g of lyophilized sourdough was weighed and put into sterile 50 mL tubes under the laminar flow cabinet, the sterile hypotonic solution was added, and cells were isolated using gradual centrifugation. After the cell collection, gDNA was extracted using a ZR Quick DNA Fungal/Bacterial Miniprep Kit (Zymo Research Corp, Irvine, CA, USA).

The quantity of the DNA was determined by Qubit™ 4 Fluorometer using a dsDNA HS Assay Kit (Thermo Fisher Scientific). All samples were qualified for sequencing (OD 260/280 > 1.8 and OD 260/230 > 1.7) and used for library preparation according to the in-house sequencing protocol [26] with V4 (F515/R806) primer pair for 16S rRNA sequencing [27], and ITS 1 (multiplex primers set) [28] and ITS2 primers for fungi identification [29] The reaction was carried out with FIREPol^®^ Master Mix (Solis BioDyne OÜ, Tartu, Estonia) in an Eppendorf Mastercycler (Sigma-Aldrich Chemie GmbH, Germany). For the multiplexing, Nextera XT Index Kit v2 Set A (Illumina Germany, Berlin, Germany) was used. PCR products were purified by NucleoMag^®^ NGS Clean-up and Size Select Magnetic Beads (MACHEREY-NAGEL, Germany) in accordance with the manufacturer’s manual.

Amplicon sequencing was performed using the iSeq 100 platform (SN FS10000643, Illumina) and iSeq 100 i1 Reagent kit v2 by 2 × 150 bp, dual index setup for 16S rRNA amplicon protocol and single-end approach for the fungal ITS pipeline.

The processing of sequencing reads according to the primer sequences has been performed with in-house scripts. 16S rRNA sequence data were analyzed using the BION-meta program [30] according to the author’s instructions. First, sequences were cleaned at both ends using a 99.5% minimum quality threshold for at least 18 of 20 bases for the 5’ end and 28 of 3’ end, then joined, followed by removal of shorter read pairs than 150 bp. Then, sequences were cleaned from chimeras and clustered by 95% oligonucleotide similarity (k-mer length of 8 bp, step size 2 bp). Lastly, consensus reads were aligned to the SILVA reference 16S rDNA database (v138) using a word length of 8 and similarity cut-off of 90%. ITS sequence data were analyzed using QIIME2 pipeline. The microbial designation was analyzed at different taxonomic levels down to species if applicable.

Statistical analysis and visualization were done in R version 4.0.2 (R Foundation for Statistical Computing, Vienna, Austria).

## 3. Results and Discussion

### 3.1. The Influence of Technological Parameters to the Development of LAB, Yeasts

#### 3.1.1. Modeling of the Influence of Variable Factors in the 1st Step

In the starting step of spontaneous fermentation, the microbiota of flour is changing and adapting to the surrounding conditions. The task of fermentation is to hinder the adverse microbiota of flour and improve the development of LAB and yeasts. In the 1st step, the development of the desired ecosystem of microorganisms is essential.

The response surface methodology with the three-factor Box–Behnken method was used to establish the optimal conditions of the fermentation of hull-less barley sourdough, together with the pH level, and lactic acid bacteria and yeasts development under the influence of the three factors (Table 2).

**Figure 1:**
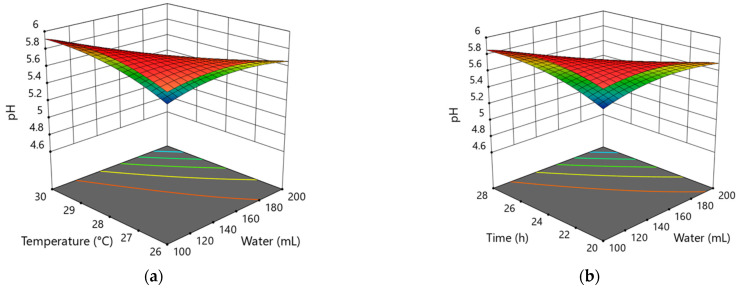

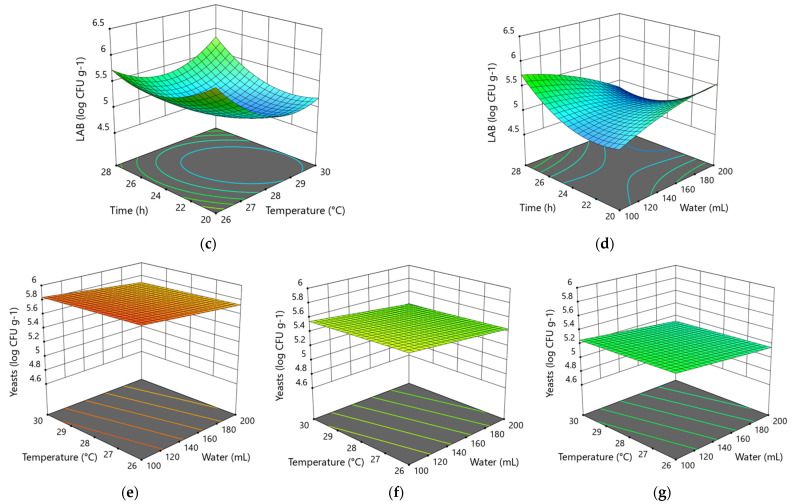
Response surface plots show the effect of temperature and water (**a**), time and water (**b**) on pH and of time and temperature (**c**), time and water (**d**) LAB log CFU g^−1^ in the 1st backslopping step of fermentation; (**e**–**g**)—shows time factor (20; 24; and 28 h) influence by yeasts log CFU g^−1^.

The results of analysis of variance results for each model are shown in Table 3. Quadratic models for pH and LAB responses were significant. The Quadratic model shows a significant influence of all dependent variables (*p* < 0.05) on the pH value (Table 3). With higher temperatures and bigger amounts of water, the level of pH reduces. During the 1st step, the lowest pH is to be expected with the maximum time of fermentation of 28 h, the maximum amount of water as 200 mL, and at 30 °C (Figure 1a). At the beginning of the fermentation process, the metabolic activity of microorganisms—lactic acid bacteria and yeasts—is low. Initially, there are processes of the activating of flour enzymes and the hydrolytic processes [25]. These processes depend on the flour and water ratio, i.e., the availability of water.

According to Table 3, the LAB quadratic model with *p* = 0.0119 shows a significant (*p* < 0.05) influence of temperature and time factors on the number of LAB in the 1st backslopping step (Figure 1b). In the 1st step, where the amount of water is between 100 and 200 mL (DY 200–400), it does not have a major influence on the development of LAB, but the mutual influence of water and time on the number of LAB is significant (*p* < 0.05). The interaction of temperature and time is also important. The optimized factors for the expected maximum number of lactic acid bacteria are 198 mL of water, the temperature of 26 °C, and the time of fermentation of 20 h.

The linear model for yeast development is not significant, with *p* = 0.0743. The increase of the number of yeasts in the 1st backslopping step is considerably (*p* < 0.05) influenced by the time factor (Figure 1c). The maximum yeast cell count can be expected during 20 h of fermentation at the temperature of 28 °C if the amount of water is 100 mL. However, the modelled parameters for the minimum number of active yeasts should be 28 h of fermentation time at 30 °C and in the presence of 200 mL of water.

The scientific literature mentions the protocols of spontaneous fermentation of rye sourdough, where the time of the 1st backslopping step fermentation is comparably long —18, 24 h (mentioned more often), or even 48 h. This period is characterized by the development of the natural diverse microbiota and low fermentation activity of lactic acid bacteria and yeasts [7]. In the research of Cakir [17], after the 24 h fermentation of the mixture of hull-less barley flour and water in the ratio 1:1 at the fermentation temperature of 28 °C, the pH decreased to 4.59. At the same time, the hull-less barley bakery sourdough production with the flour–water ratio of 1:1 and the temperature of 17–22 °C showed no changes in observed pH levels after 24 h of fermentation, meaning there was very low metabolic activity. In phase 1, the fermented mixture of water and flour can be considered as an unripe or newborn sourdough [18].

The basis for the selection of the technological parameters of the study was to obtain a sourdough of type 1 and secure the leavening ability of the sourdough, i.e., maximum preserving of the symbiosis of lactic acid bacteria and yeasts. The optimized parameters obtained in the model in the 1st phase and chosen for preparation of the wholemeal hull-less barley sourdough are 24 h of fermentation with 113 mL of water at 30 °C.

#### 3.1.2. Modeling of the Influence of Variable Factors in the 2nd Backslopping Step

In the second phase, it is important to develop the lactic acid bacteria for the continuing lowering of acidity. The design and results of Box–Behnken modelling are represented in the table with the responses for pH levels, and LAB and yeasts enumeration (Table 4).

**Figure 2:**
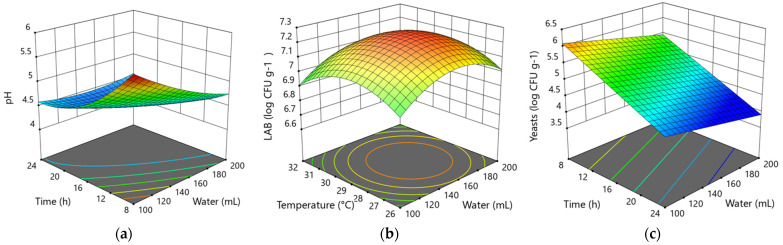
Response surface plots show the interaction effect of parameters on pH (**a**), LAB (**b**) log CFU g^−1^ and yeasts log CFU g^−1^ (**c**) in the 2nd backslopping step of 14 h fermentation.

In the 2nd backslopping step, the pH, LAB and yeast models are significant (Table 3). The Quadratic model of pH shows meaningful interaction between time and water parameters (Figure 2a). The highest value of pH is detected with the 8 h of fermentation and 100 mL of added water. However, the most rapid increase of acidity until pH 4.5 is observed at the maximum amount of water of 200 mL and with the maximum time of fermentation of 24 h at 32 °C.

However, the 2FI (two-factor interaction) LAB model with *p* = 0.0445 shows a significant interaction of time and water amount with (*p* = 0.0131). Response surface plots (Figure 2b) show the impact of time and water on LAB counting. The modelled factors for the maximum count of lactic acid bacteria are identified as 24 h of fermentation with 190 mL of added water at 34 °C.

The count of yeasts colony-forming units is significantly influenced by the factor of the time (Figure 2c), since yeasts are sensitive to low acidity. The lowest acidity is observed with the longest time of fermentation; therefore, the time has a substantial impact on the development of yeasts. The maximum increase of yeast CFU in this model is expected during 8 h of fermentation, 100 mL of water at 29 °C.

For the optimizing of variable factors, the criteria with the maximum amount of lactic acid bacteria and yeasts were chosen. The optimized factors for such criteria are 14 h of fermentation time with 137 mL of added water and 31 °C.

#### 3.1.3. Modeling of the Influence of Variable Factors in the 3rd Backslopping Step

The effect of three influencing factors on pH, lactic acid bacteria and yeasts is presented in Table 5. The goal of the 3rd backslopping step is to achieve the counts of lactic acid bacteria and yeast CFU that are characteristic of active sourdough. Usually, the 3rd backslopping step is characterized by a group of acid-sensitive bacteria including *Pediococcus* and *Latilactobacillus* [18].

In the 3rd phase, the Quadratic models do not show a significant (*p* > 0.05) influence of the variable factors on any studied parameters. The development of lactic acid bacteria and yeasts in this phase shows stable growth, and the factors within the limits of this model do not significantly influence their development. The maximum yeast cell count can be expected during 15 h of fermentation at the temperature of 26 °C if the amount of water is 100 mL, but the maximum LAB count developed at the temperature 29 °C during 10 h fermentation and 153 mL of added water.

**Figure 3:**
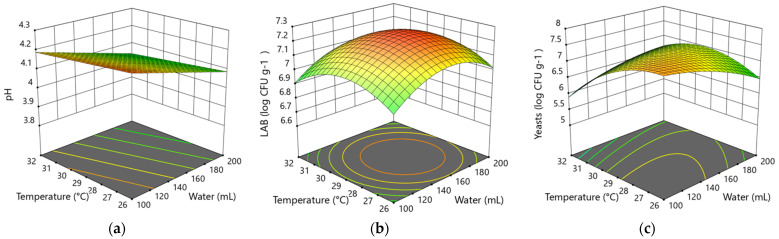
Response surface plots show the effect of temperature and water on pH (**a**), LAB (**b**) and yeasts (**c**) log CFU g^−1^ in the 3rd backslopping step of fermentation at the actual factor (12 h).

The optimized factors for the maximum amount of LAB and yeasts in the 3rd backslopping step are 12 h fermentation time with the amount of added water 153 mL and temperature 28.5 °C. The interaction effect of temperature and water on pH (**a**), LAB (**b**) and yeasts (**c**) at the 12 h fermentation time is shown in Figure 3.

### 3.2. The Dynamics of the Development of Lactic Acid Bacteria and Yeasts in Three-Step Fermentation According to the Optimized Factors

To check the model, three-step fermentation of hull-less barley sourdough was done according to the optimized influencing parameters (Table 6).

Optimized temperature and time parameters coincide with wheat and rye sourdough fermentation conditions described in the literature. Similar to this research, the French and American systems require 24 h fermentation in the first backslopping step. On the other hand, the length of the second and third backslopping steps in the previously mentioned systems is 8 and 16 h. Even though the standard spontaneous or type 1 sourdough fermentation has a temperature of 30 °C, the American spontaneous fermentation system requires 32–35 °C in all three steps of fermentation. Our research indicates the highest optimal temperature is 31 °C in the 2nd backslopping step. The optimized ratio of flour and amount of added water in each backslopping step is different. In the American system, the ratio of flour and added water is 1:1.5 (DY = 250) and this corresponds to the ratio of flour and water in the 3rd backslopping of our study. However, this parameter is higher than the typical type 1 of spontaneous fermentation.

This study experimentally determined that at the beginning of the first fermentation step, the LAB amount is 3.1 log CFU g^−1^, but the yeasts is 4.9 log CFU g^−1^. As the obtained results show, it is possible to develop the count of lactic acid bacteria in the controlled environment of three phases of spontaneous fermentation that characterizes an active sourdough: 3.1 log CFU g^−1^ at the start until 8.2 log CFU g^−1^ at the end of 3rd backslopping, and the number of yeasts from 4.9 to 7.3 log CFU g^−1^ at the end of the 3rd backslopping (Table 6). Besides, the pH change of the sourdough from 5.4 after the 1st backslopping step to 3.8 at the end of fermentation shows the metabolic activity of LAB (Table 6).

### 3.3. Identification of Microorganisms in the Hull-Less Barley Sourdough in the Three Backslopping Steps of Fermentation

The diverse microbiota of wholegrain hull-less barley flour in a controlled environment can be directed towards the development of desired microorganisms. It was confirmed by previously acquired research aimed at the identification of microorganisms. To explore the diversity of the microbiota of sourdough, the isolates were analyzed using the amplicon next-generation sequencing. The diversity of microorganisms was established in wholegrain flour of hull-less barley as well as during three backslopping steps of fermentation. Hull-less barley flour (HBF; HBF.ITS1; HBF.ITS2) and sourdough samples were analyzed at the end of the 1st backslopping step (HBS1; HBS1.ITS1; HBS1.ITS2), 2nd backslopping step (HBS2; HBS2.ITS1; HBS2.ITS2), and at the final point of fermentation (HBS3; HBS3.ITS1; HBS3.ITS2) by 16S rRNA and ITS sequencing methodology for bacterial and fungal consortia correspondently.

**Figure 4:**
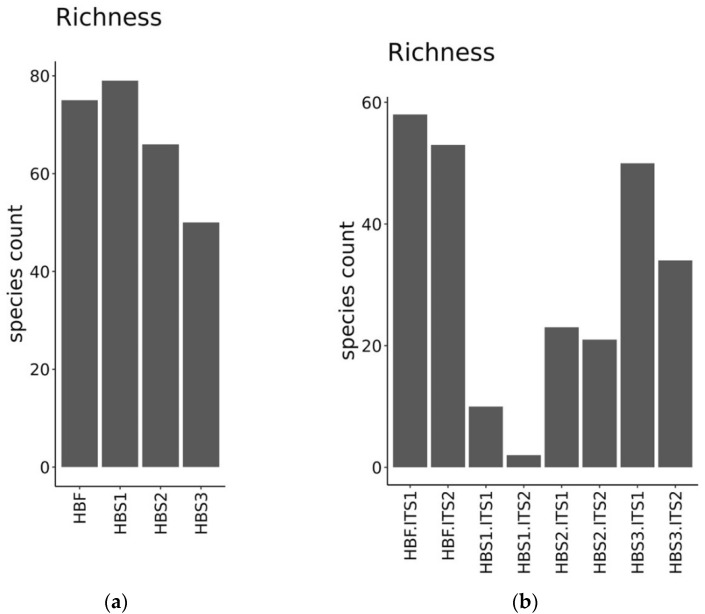
Richness plot (**a**) shows richness of bacteria in hull-less barley flour (HBF) and in hull-less barley sourdough at the end of the 1st backslopping step (HBS1), 2nd backslopping step (HBS2) and 3rd backslopping step (HBS3) of fermentation. Plot (**b**) shows the richness of fungi in hull-less barley flour (HBF.ITS1; HBF.ITS2) and in hull-less barley sourdough at the end of the 1st backslopping step (HBS1.ITS1; HBS1.ITS2); 2nd backslopping step (HBS2.ITS1; HBS2.ITS2) and 3rd backslopping step (HBS3.ITS1; HBS3.ITS2) of fermentation.

In the richness plot, the number of identified species is expressed as species count and shows the diverse microbiota presence in the hull-less barley flour and at the end of every fermentation phase (Figure 4). The highest numbers of bacteria species (more than 70) were identified in the sample of sourdough at the end of the 1st backslopping step (Figure 4a). As shown in Figure 4b, the largest fungi species count was found in hull-less barley flour HBF.ITS1 and HBF.ITS2, but the smallest count was found in the sample after the 1st fermentation phase.

**Figure 5:**
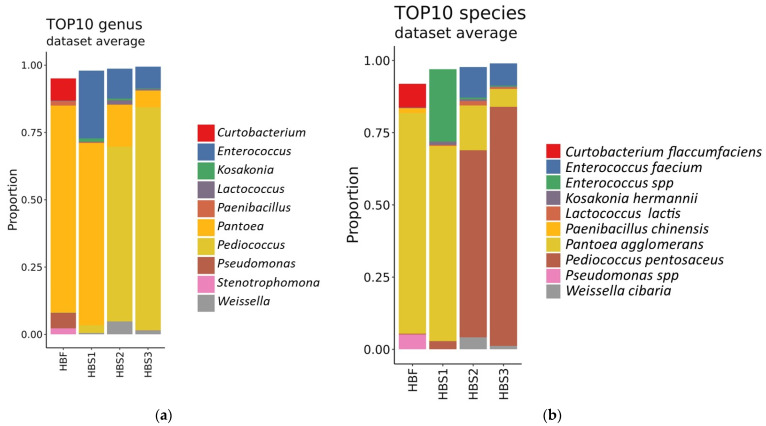
Taxonomic composition of the 10 most represented for selected set of bacteria. (**a**)—The composition of the most represented bacteria genus for hull-less barley flour HBF and the sourdough after the 1st, 2nd, 3rd backslopping step of fermentation (HBS1; HBS2, HBS3); (**b**)—the composition of the most represented bacteria species for hull-less barley flour HBF and for the sourdough after the 1st, 2nd, 3rd phase of fermentation (HBS1; HBS2, HBS3).

The taxonomic composition of the 10 most represented bacteria genus for each sample are shown in Figure 5a. Composition of bacteria that were proportionally found in the largest quantities in wholegrain flour of hull-less barley were *Pantoea*, *Curtobacterium* and *Pseudomonas* (Figure 5a) with the most represented species such as *Pantoea agglomerans*, *Curtobacterium flaccumfaciens*, and *Pseudomonas* spp. (Figure 5b). During the fermentation stage, the balance in bacteria shifts to *Pediococcus pentosaceus* prevalence.

To identify the majority of fungi in the samples, two parallel protocols utilizing ITS1 and ITS2 fungal regions were chosen. This approach helps to overcome technological limitations connected with the single sequencing strategy and identify more fungal species.

**Figure 6:**
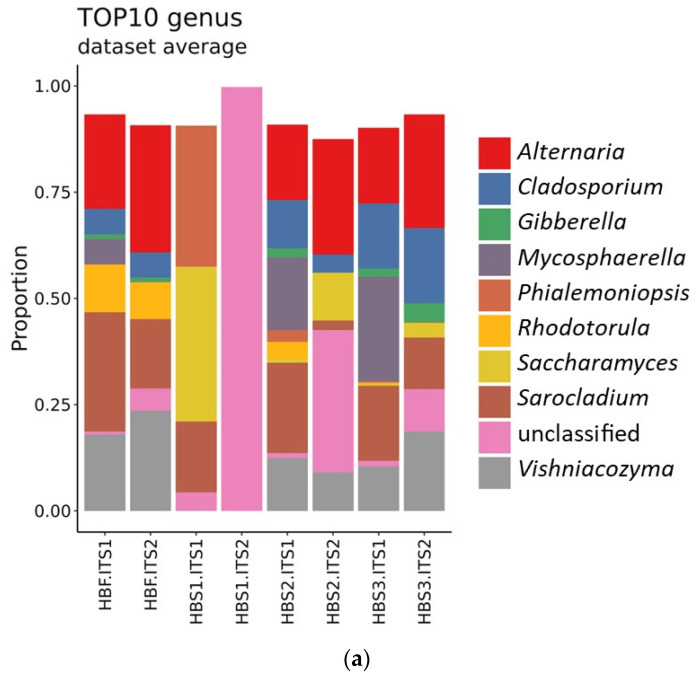

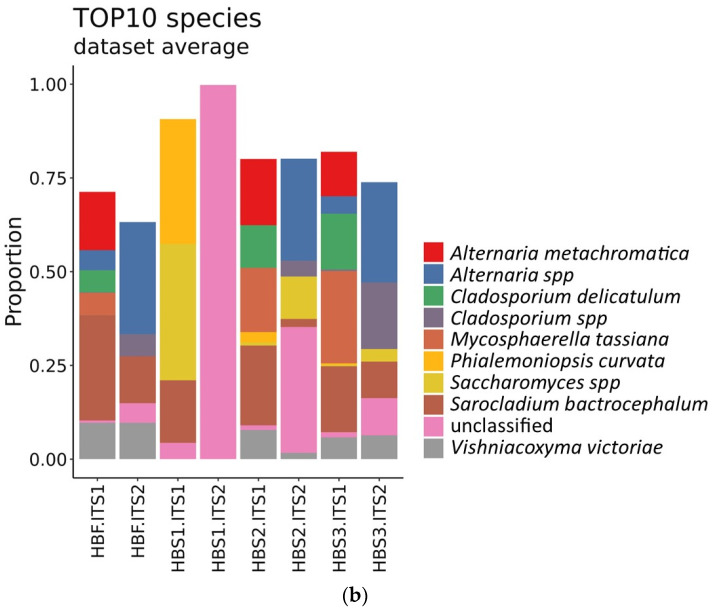
Taxonomic composition of the 10 most represented fungi genus (**a**) and species (**b**) for hull-less barley flour and in the sourdough after the 1st, HBS1.ITS1; HBS1.ITS2; 2nd, HBS2.ITS1; HBS2.ITS2, and the 3rd HBS3.ITS1; HBS3.ITS2 backslopping steps of fermentation.

The dominating fungal genera detected in the samples were *Vishniacozyma, Sarocladium, Alternaria* and *Cladosporium*. Similar results are described by Katsi [31] study on wheat sourdough. Among the prevalent species of the fungi in the flour were *Alternaria* spp., *Cladosporium delicatulum, Mycosphaerella tassiana, Sarocladium bactrocephalum*, and *Vishniacozyma victoriae* (Figure 6). The fermentation process adds *Phialemoniopsis curvata* and *Saccharomyces* spp. to the samples. It is interesting that by analogy with bacteria, the first stage of fermentation brings the most drastic microbiota changes.

The above-mentioned species create the natural, typical microbiota of the cereal. Other scientific studies have also pointed to the diverse microbiota of flour [32] and sourdoughs [6]. Among others, the specific structure of the grain also contributes to the diversity of microbiota of the wholegrain flour of hull-less barley. The grain of hull-less barley does not have the hull, which is separated during the processing, and is the common environment for microorganism growth. This defines the microbiota of hull-less barley and impacts the fermentation.

In the sourdough after the 1st backslopping step of fermentation (sample HBS1), the dominating species are *Pantoea agglomerans* and *Enterococcus faecium*, and their proportions after 2nd and 3rd backslopping step (samples HBS2 and HBS3) have decreased (Figure 5b). In the 2nd and 3rd steps, the dominating development of *Pediococcus pentosaceus* species was observed, followed by *Pantoea agglomerans*, *Enterococcus faecium*, *Weisella cibaria*, and *Lactococcus lactis* presence. As mentioned in the scientific literature, *Pediococcus* and *Weisella* species are less dominating in the typical rye and wheat sourdoughs, however, in the barley sourdoughs, *Pediococcus* species have been found as dominating [17]. In addition, homofermentative species of *Enterococcus*, *Lactococcus* and *Streptococcus* usually are subdominant in sourdoughs [7,18,33] and are found in the initial stages of sourdough fermentation after the 24 h of fermentation and the first three refreshments [34]. Initially, these non-specific lactic acid bacteria can be important for rapid acidification of water–flour mixture and thereby activate the enzymes present in the flour [8]. They are stronger acidifiers compared to the typical lactic acid bacteria and create a favorable environment for the establishment of typical sourdough species. These subdominant specific species are suitable or have adapted to grow in the materials of a plant origin [34]. In this study, highly adapted LAB species were not found after the 3rd backslopping step. However, the prevalence of highly adapted LAB species (e.g., *Lactobacillus plantarum*, *Lactobacillus fermentum* [35]; new names—*Lactiplantibacillus plantarum*, *Limosilactobacillus fermentum* [16,18]) after 5–7 backslopping steps is observed in other studies.

The developmental changes of microscopic fungi species in the 2nd backslopping step of fermentation show the dominance of *Sarociadum bactrocephalum, Alternaria*, and *Saccharomyces* spp., but after the 3rd backslopping step of fermentation—*Alternaria* and *Clodosporium* spp. Sourdoughs with the dominating natural microbiota of the original grains and lactic acid bacteria that are not typical for the sourdoughs are considered as young or immature (not ripe). Our results confirm the dynamic development of the community of lactic acid bacteria during the fermentation process of hull-less barley sourdough.

To get the whole picture and the understanding of the fermentation process, it is necessary to perform further experiments with wholegrain hull-less barley sourdough in several generations and to analyze the impact of fermentation factors not only on the community of lactic acid bacteria but also on the development of specific species of these bacteria in ripe sourdoughs. It would be worthwhile to explore the evolution of *Pediococcus pentosaceus* in ripe spontaneous sourdoughs of hull-less barley to use the anti-microbial, antifungal and phytase activities of this species [17,36] and the ability to separate peptidases for splitting the allergic peptides [37].

## 4. Conclusions

Our study showed the significant influence of the amount of the added water, fermentation temperature and time on the pH level and the microbiological parameters of the wholegrain hull-less barley sourdough. Created models of the final fermentation stage can be used to generate the technological process of spontaneous sourdough fermentation and achieve the necessary quality of the sourdough.

The usage of wholegrain hull-less barley flour in the fermentation of sourdoughs leads to the distinct microbiota. In the controlled spontaneous three-step backslopping fermentation environment, the hull-less barley sourdough at the end of the fermentation process shows the dominance of *Pediococcus pentosaceus*, and the presence of *Weisella cibaria* and *Lactococcus lactis* bacteria species. Moreover, we detected the diverse fungal sourdough community. During the fermentation process, the typical adverse microbiota of grains is reduced, thereby securing an appropriate quality of sourdough to produce wholegrain bread. As the result, an active hull-less barley sourdough has been obtained.

## Figures and Tables

**Table 1:** The three independent variables/parameters and levels.

Independent Variables	Units	Symbols	Coded Levels								
			1st Backslopping Step			2nd Backslopping Step			3rd Backslopping Step		
			−1	0	1	−1	0	1	−1	0	1
Amount of added water	mL	X_1_	100	150	200	100	150	200	100	150	200
Temperature of fermentation	°C	X_2_	26	28	30	29	32	35	26	29	32
Time of fermentation	h	X_3_	20	24	28	8	16	24	8	14	20

**Table 2:** The Box–Behnken experimental design and responses data after the 1st backslopping step of fermentation.

1st Backslopping Step						
	Independent Variables			Responses		
Run	X_1_	X_2_	X_3_	pH	LAB	Yeasts
	Water, mL	Temperature, °C	Time, h		log CFU g^−1^	log CFU g^−1^
1	100	26	24	5.84 ± 0.09	5.50 ± 0.31	5.89 ± 0.09
2	200	26	24	5.72 ± 0.00	5.29 ± 0.05	5.34 ± 0.33
3	100	30	24	5.86 ± 0.02	5.27 ± 0.31	5.74 ± 0.22
4	200	30	24	4.62 ± 0.09	5.02 ± 0.02	5.04 ± 0.04
5	100	28	20	5.82 ± 0.05	4.93 ± 0.14	5.80 ± 0.08
6	200	28	20	5.72 ± 0.00	5.58 ± 0.41	5.86 ± 0.09
7	100	28	28	5.83 ± 0.02	5.48 ± 0.27	4.70 ± 0.11
8	200	28	28	4.68 ± 0.00	4.69 ± 0.41	5.33 ± 0.32
9	150	26	20	5.77 ± 0.00	6.50 ± 0.26	5.58 ± 0.25
10	150	30	20	5.72 ± 0.02	5.48 ± 0.23	5.95 ± 0.04
11	150	26	28	5.50 ± 0.13	5.45 ± 0.22	5.45 ± 0.28
12	150	30	28	5.03 ± 0.04	5.39 ± 0.08	5.39 ± 0.23
13	150	28	24	5.70 ± 0.07	4.97 ± 0.13	5.41 ± 0.03
14	150	28	24	5.75 ± 0.00	4.86 ± 0.54	5.54 ± 0.06
15	150	28	24	5.70 ± 0.09	5.01 ± 0.21	5.42 ± 0.09

**Table 3:** Analysis of variance of the models.

Responses	Effect of Factors in the Models										
	Source	Model	X_1_	X_2_	X_3_	X_1_X_2_	X_1_X_3_	X_2_X_3_	X_1_^2^	X_2_^2^	X_3_^2^
1st step											
pH	*p*-value	0.0007	0.0002	0.0017	0.0006	0.0018	0.0023	0.0720	0.0850	0.0699	0.0746
	Coef	5.72	−0.3263	−0.2000	−0.2487	−0.2800	−0.2625	−0.1050	−0.1029	−0.1104	−0.1079
	R^2^	0.9826									
LAB	*p*-value	0.0119	0.2708	0.0225	0.0283	0.9117	0.0085	0.0380	0.2888	0.0048	0.0141
	Coef	4.95	−0.0750	−0.1975	−0.1850	−0.0100	−0.3600	0.2400	−0.1058	0.4292	0.3292
	R^2^	0.9441									
Yeasts	*p*-value	0.0743	0.4937	0.8627	0.0136						
	Coef	5.50	0.4937	0.8627	0.0136						
	R^2^	0.4537									
2nd step											
pH	*p*-value	0.0009	0.0004	0.5801	0.0002	0.6013	0.0025	0.0818	0.0636	0.0953	0.0010
	Coef	4.47	−0.2687	−0.0188	−0.3150	−0.0250	0.2525	0.0975	0.1108	0.0958	0.3233
	R^2^	0.9812									
LAB	*p*-value	0.0445	0.6672	0.2348	0.3794	0.6056	0.0131	0.0902			
	Coef	5.45	0.0972	0.1262	0.0844	0.0431	0.1909	0.0772			
	R^2^	0.7376									
Yeasts	*p*-value	0.0140	0.1612	0.3647	0.0035						
	Coef	5.00	−0.3150	−0.1988	−0.7763						
	R^2^	0.6039									
3rd step											
pH	*p*-value	0.1163	0.0441	0.4796	0.2170						
	Coef	4.12	−0.0737	−0.0237	−0.0425						
	R^2^	0.4026									
LAB	*p*-value	0.1908	0.3008	0.7747	0.0323	1.0000	0.4724	0.8536	0.0821	0.0946	0.2037
	Coef	7.21	0.0525	−0.0138	−0.1338	0.0000	0.0500	0.0125	−0.1454	−0.1379	−0.0979
	R^2^	0.8029									
Yeasts	*p*-value	0.2109	0.7649	0.1490	0.9053	0.1876	0.4292	0.8290	0.6004	0.2224	0.0191
	Coef	7.24	−0.0663	−0.3575	−0.0263	0.4525	−0.2550	−0.0675	−0.1725	−0.4300	−1.05
	R^2^	0.7925									

X_1_-water; X_2_-temperature; X_3_-time are factors (parameters); Coef-coefficient estimate of factors; responses—pH, lactic acid bacteria log CFU g^−1^ (LAB), yeasts log CFU g^−1^ (Yeasts) after the 1st, 2nd and 3rd backslopping step. In the 1st step for pH and LAB quadratic model, for yeasts—linear model; in the 2nd step for pH-quadratic model, for LAB-2F1 (two-factor interaction), for yeast-linear model; in the 3rd step for the pH linear model; for LAB and yeasts, quadratic models are shown.

**Table 4:** The Box–Behnken experimental design and responses in the 2nd backslopping step of fermentation.

2nd Backslopping Step						
	Independent Variables			Responses		
Run	X_1_	X_2_	X_3_	pH	LAB	Yeasts
	Water, mL	Temperature, °C	Time, h		log CFU g^−1^	log CFU g^−1^
1	100	29	16	5.02 ± 0.02	5.98 ± 0.39	6.26 ± 0.08
2	200	29	16	4.44 ± 0.02	5.48 ± 0.08	4.27 ± 0.07
3	100	35	16	4.97 ± 0.03	5.79 ± 0.04	4.57 ± 0.29
4	200	35	16	4.29 ± 0.01	5.55 ± 0.06	4.35 ± 0.09
5	100	32	8	5.71 ± 0.02	5.78 ± 0.07	6.11 ± 0.04
6	200	32	8	4.76 ± 0.01	4.60 ± 0.07	5.62 ± 0.12
7	100	32	24	4.55 ± 0.03	5.58 ± 0.13	4.53 ± 0.22
8	200	32	24	4.61 ± 0.01	5.93 ± 0.10	4.71 ± 0.08
9	150	29	8	5.28 ± 0.04	5.96 ± 0.27	6.35 ± 0.26
10	150	35	8	5.11 ± 0.01	5.39 ± 0.05	6.21 ± 0.04
11	150	29	24	4.48 ± 0.01	5.49 ± 0.08	4.34 ± 0.09
12	150	35	24	4.70 ± 0.02	5.84 ± 0.09	4.50 ± 0.04
13	150	32	16	4.43 ± 0.03	5.32 ± 0.16	4.41 ± 0.07
14	150	32	16	4.42 ± 0.02	5.29 ± 0.11	4.30 ± 0.09
15	150	32	16	4.57 ± 0.02	5.46 ± 0.09	4.40 ± 0.11

**Table 5:** The Box–Behnken experimental design and responses in the 3rd backslopping step of fermentation.

3rd Backslopping Step						
	Independent Variables			Responses		
Run	X_1_	X_2_	X_3_	pH	LAB	Yeasts
	Water, mL	Temperature, °C	Time, h		log CFU g^−1^	log CFU g^−1^
1	100	26	14	4.27 ± 0.01	6.98 ± 0.08	7.05 ± 0.21
2	200	26	14	4.13 ± 0.01	7.03 ±0.39	6.45 ± 0.26
3	100	32	14	4.24 ± 0.02	6.83 ± 0.18	5.92 ± 0.50
4	200	32	14	4.06 ± 0.03	6.88 ± 0.08	7.13 ± 0.07
5	100	29	8	4.22 ± 0.02	7.14 ± 0.14	6.17 ± 0.14
6	200	29	8	4.09 ± 0.00	7.20 ± 0.10	6.11 ± 0.10
7	100	29	20	4.01 ± 0.04	6.64 ± 0.32	6.43 ± 0.10
8	200	29	20	3.87 ± 0.03	6.90 ± 0.13	5.35 ± 0.13
9	150	26	8	4.15 ± 0.03	7.01 ± 0.08	6.22 ± 0.27
10	150	32	8	4.08 ± 0.00	7.08 ± 0.15	5.15 ± 0.09
11	150	26	20	4.17 ± 0.01	6.85 ± 0.07	6.50 ± 0.11
12	150	32	20	4.15 ± 0.03	6.97 ± 0.15	5.16 ± 0.08
13	150	29	14	4.25 ± 0.02	7.13 ± 0.09	7.92 ± 0.07
14	150	29	14	4.13 ± 0.01	7.23 ± 0.05	7.15 ± 0.09
15	150	29	14	4.04 ± 0.01	7.80 ± 0.06	6.65 ± 0.14

**Table 6:** Optimized factors in the three backslopping steps of fermentation and experimentally determined characteristic parameters: pH, LAB and yeasts enumeration.

Backslopping Step	Parameters			Predicted Values			Experimental Values		
	Water	Temperature	Time	pH	LAB	Yeasts	pH	LAB	Yeasts
	mL	°C	h		log CFU g^−1^			log CFU g^−1^	
1st	113	30	24	5.7	5.3	5.5	5.44 ± 0.04a	5.85 ± 0.50b	6.75 ± 0.47a
2nd	137	31	14	4.7	5.6	5.3	4.62 ± 0.05b	5.99 ± 0.44b	6.86 ± 0.04a
3rd	150	28.5	12	4.1	7.2	6.8	3.80 ± 0.05c	8.22 ± 0.14a	7.34 ± 0.68a

Values with different superscript letters in the same column are significantly different (*p* < 0.05) as analyzed by Tukey test.

## Data Availability

The data is available on request.
